# Hempseeds as sustainable plant-based source of bioactive peptides with biofunctional properties

**DOI:** 10.3389/fnut.2026.1926667

**Published:** 2026-09-23

**Authors:** Anh T. L. Nguyen, Charles F. Manful, Alberta N. A. Aryee

**Affiliations:** 1 Food Science & Biotechnology Program, Department of Human Ecology, College Agriculture, Science and Technology, Delaware State University, Dover, DE, United States; 2 School of Science and the Environment, Grenfell Campus, Memorial University of Newfoundland, Corner Brook, NL, Canada

**Keywords:** bioactive peptides, bioavailability, hempseeds, inflammation, processing

## Abstract

The contribution of oxidative stress and inflammation to various diseases coupled with the many side-effects of pharmaceuticals has underscored the need for strategies that integrate multimodal approaches involving interventions that naturally balance redox homeostasis and lower chronic inflammation. This includes bioactive agents such as plant-derived bioactive peptides (BAPs) that provide physiological benefits beyond their nutritional value. BAPs are short functional amino acid (AA) sequences, typically 2–20 residues, released from proteins during food processing or GI digestion, and may act as natural antioxidants and signaling modulators. Although widely evaluated in traditional plant protein sources, BAPs are increasingly studied in non-conventional sources such as hempseed (*Cannabis sativa* L.), a sustainable oilseed rich in nutrients and whose major storage proteins include edestin and the water-soluble albumin fraction. This review summarizes how hempseed composition and processing influence protein accessibility, AA profile, peptide release, post-digestion bioactivity, and bioavailability, providing insights into the development of hempseed-derived peptides as sustainable bioactive ingredients, supporting their further development in functional foods and nutraceutical products targeting oxidative stress and inflammation.

## Graphical abstract

**Figure:**
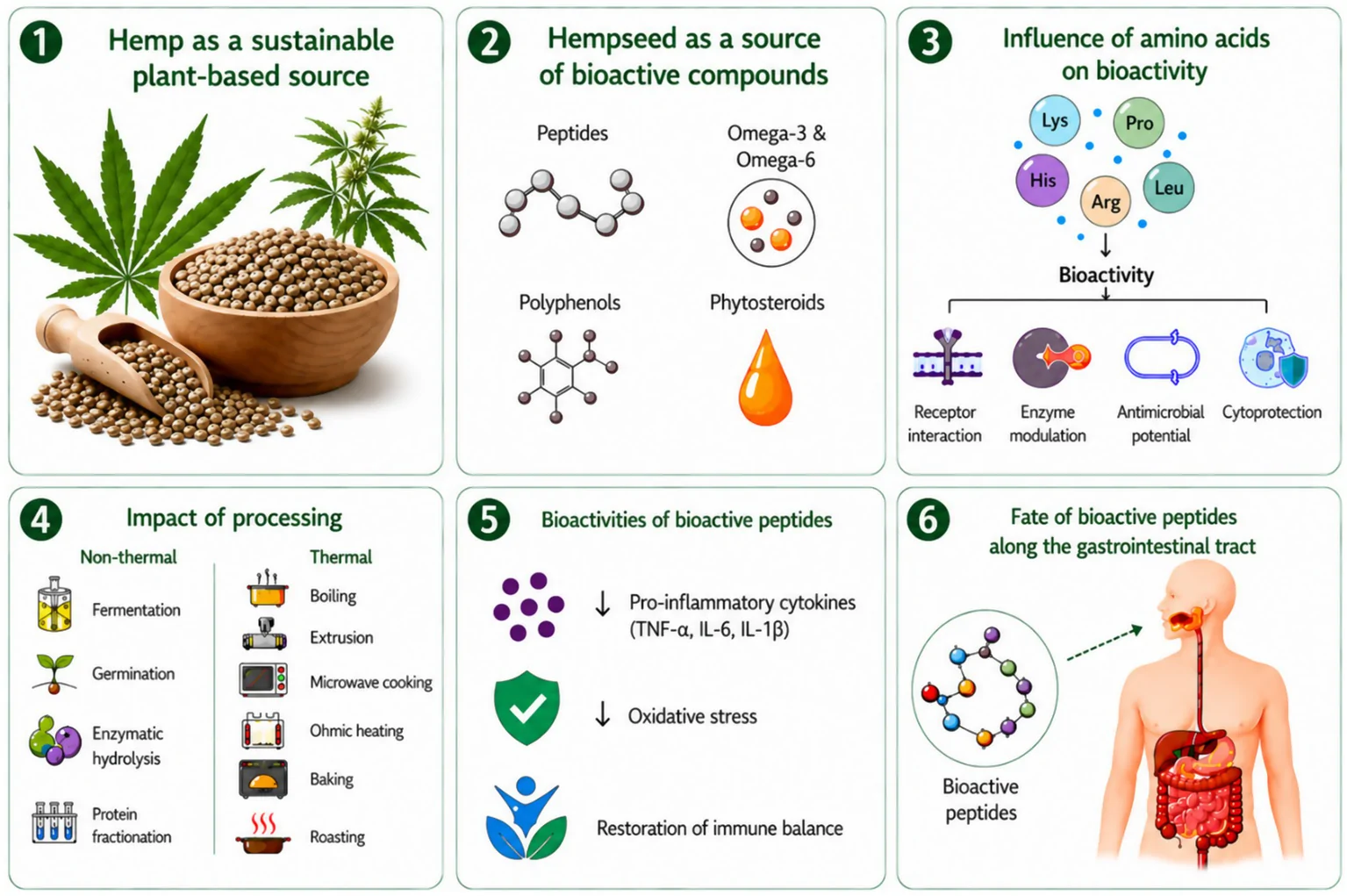

## 1 Introduction

### 1.1 Oxidative stress and chronic inflammation as therapeutic targets

The role of oxidative stress and inflammation in the initiation and progression of chronic diseases such as obesity, insulin resistance, cancer, neurological disorders, and cardiovascular and autoimmune diseases is well documented (Figure 1) (1–3). Oxidative stress occurs when reactive oxygen species (ROS) overwhelm endogenous antioxidant defenses, resulting in lipid peroxidation, protein oxidation, DNA damage, and cellular dysfunction (1, 2, 4). Although ROS contribute to cellular signaling at physiological levels, excessive ROS can activate redox-sensitive inflammatory pathways, including nuclear factor-kappa B (NF-κB) and mitogen-activated protein kinase (MAPK), thereby increasing cytokine, chemokine, inducible nitric oxide synthase (iNOS), and cyclooxygenase-2 (COX-2) expression (1, 2). Inflammation is a protective immune response to infection, injury, toxins, or cellular stress; however, unresolved or persistent inflammation promotes continued production of reactive species and inflammatory mediators, contributing to tissue damage and disease progression (2, 4, 5). Macrophages are central to this response as inflammatory stimuli, such as lipopolysaccharide (LPS), induce the production of nitric oxide (NO), tumor necrosis factor-alpha (TNF-α), interleukin-6 (IL-6), interleukin-1 beta (IL-1β), iNOS, and COX-2 (2, 4, 5). Oxidative stress and inflammation therefore reinforce each other: excess ROS activates inflammatory signaling, while activated immune cells generate additional ROS (1, 2). This interaction is especially relevant in obesity-related inflammation, where metabolic stress promotes immune-cell infiltration and cytokine production in adipose tissue, contributing to systemic inflammatory and oxidative dysregulation (3).

**Figure 1:**
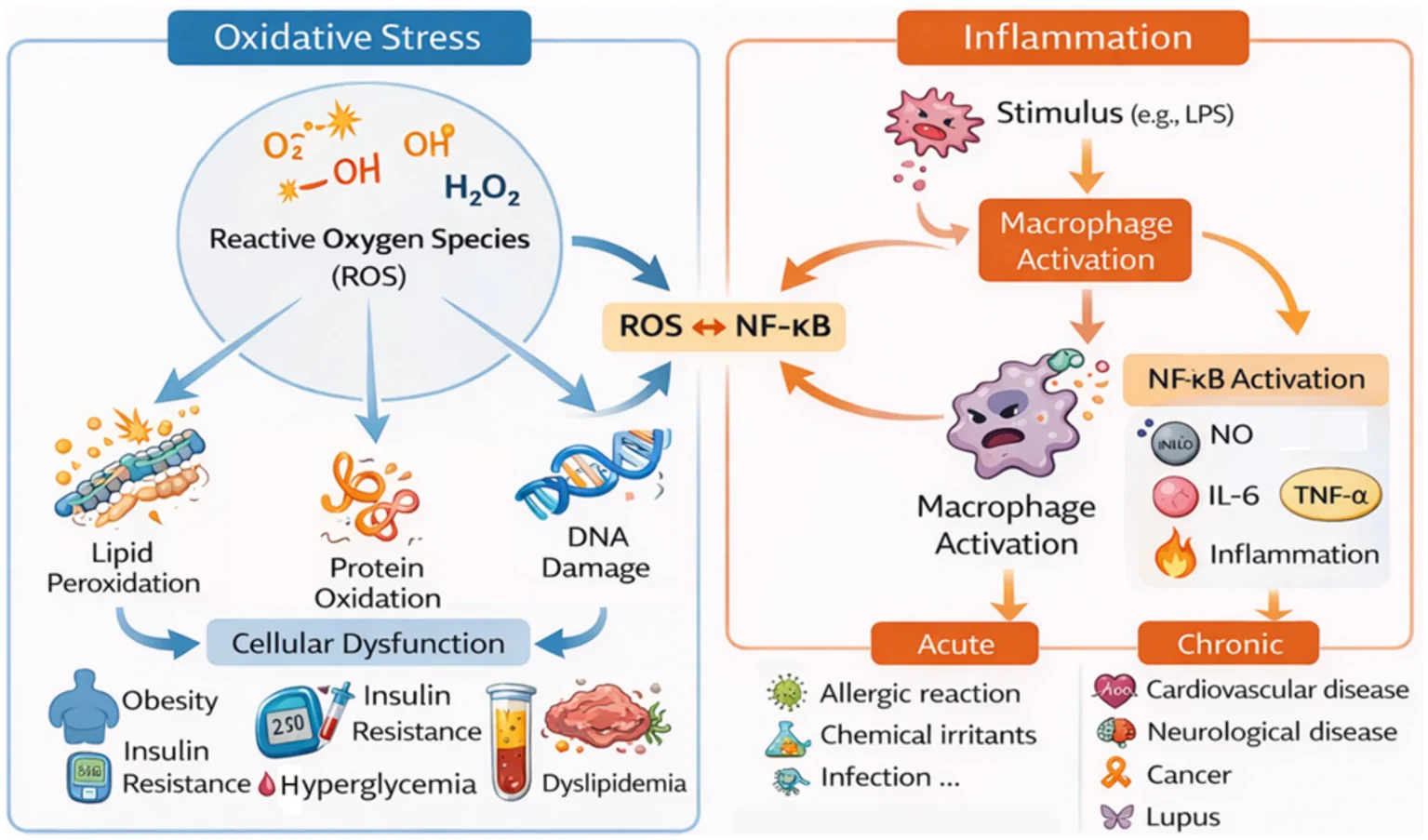
Mechanisms of oxidative stress and inflammation.

Although pharmacological interventions, including drugs and therapies, can manage inflammation and oxidative stress, their long-term use may be limited by adverse effects, inconsistent efficacy, and treatment burden (6, 7), establishing the therapeutic rationale for dietary and food-derived interventions. Among candidate dietary sources, hemp (*Cannabis sativa* L.) has drawn growing interest, though its industrial and nutritional use is often confused with drug-type cannabis. Industrial hemp refers to low-THC varieties cultivated for fiber and seed, legally distinguished from marijuana by regulatory THC thresholds, most widely set at <0.3% in jurisdictions such as the United States, European Union, and Canada; however, this threshold is not a single universal standard, as regulatory limits and enforcement vary by jurisdiction (see Section 2.6, ‘Current market status, technical challenges, and future perspectives,’ for further discussion of international regulatory inconsistency) (8). Clarifying this distinction upfront situates hempseed-derived ingredients discussed later in this review as food-grade rather than psychoactive material.

### 1.2 Plant-derived bioactive peptides as dietary interventions

Plant proteins serve as important precursors for bioactive peptides (BAPs), short amino acid sequences released during processing or GI digestion that can act as natural antioxidants, ROS scavengers, and signaling modulators of pro-inflammatory pathways (9, 10). Therefore, their bioactivity depends not only on their presence in the food matrix, but also on peptide formation, amino acid (AA) sequence characteristics, stability during digestion, and bioavailability (9–12). They may offer advantages including compatibility with functional food formulations and consumer preference for naturally derived ingredients (10, 13). Food-derived BAPs are short functional amino acid (AA) sequences, typically containing 2–20 residues (10, 13). These short, low MW (LMW) BAPs display multifaceted bioactivities including antioxidant and anti-inflammatory properties by scavenging free radicals, chelating metal ions, and donating electrons or hydrogen atoms, which may help reduce oxidative damage (Figure 2) (12, 14, 15). BAPs may reduce inflammatory mediator production via the redox-sensitive pathways described above, as well as through Nrf2/HO-1 signaling (2, 5, 12, 16). Their bioactivities depend on peptide sequence, individual AA and groups of AA (hydrophobic, aromatic, sulfur-containing, or positively charged AA), residue position, MW, stability, and bioavailability (12, 16).

**Figure 2:**
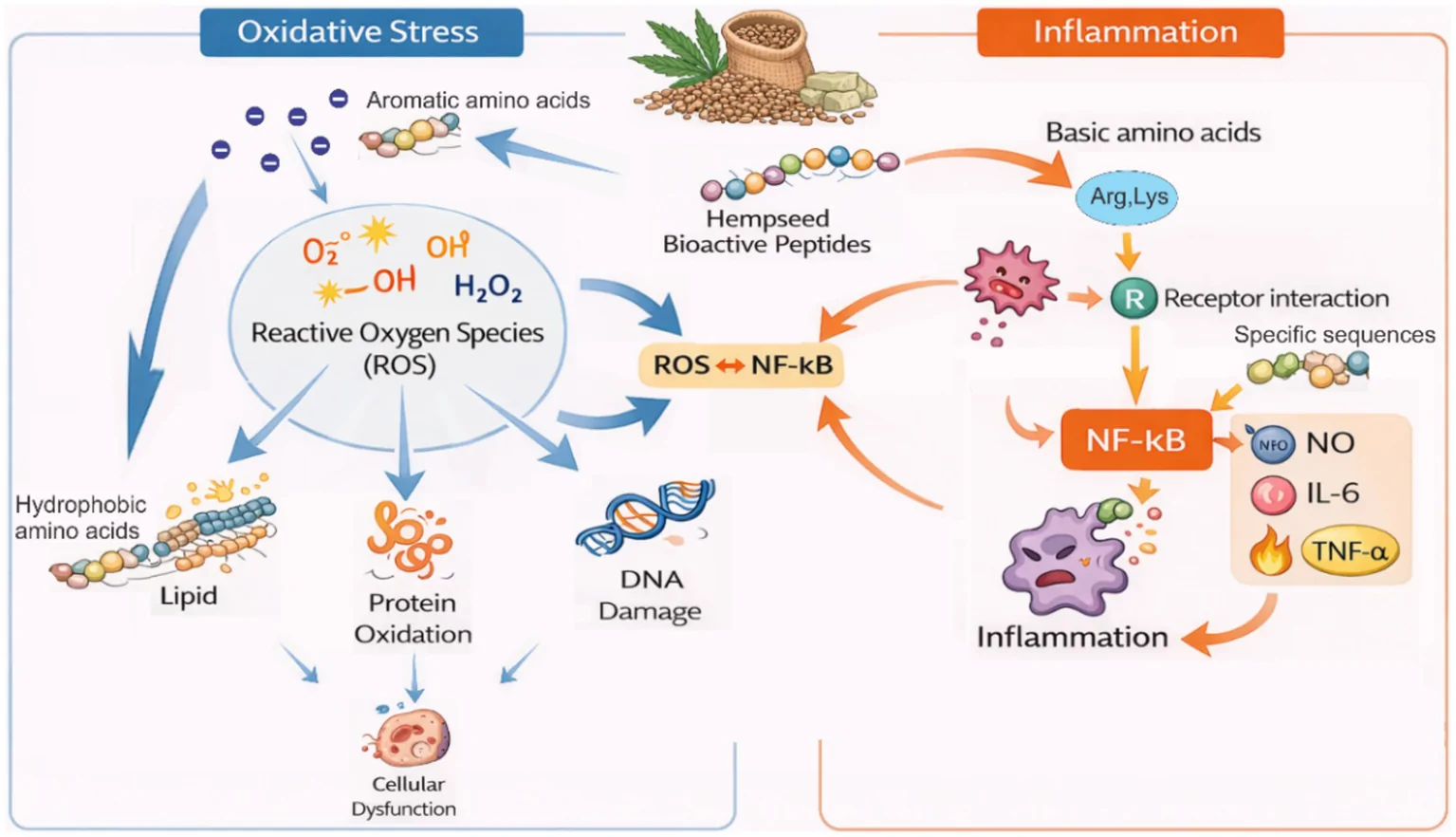
Mechanism of hempseed bioactive peptides in oxidative stress and inflammation.

Demand for sustainable, high-quality protein has increased and food systems have shifted toward plant-based sources with combined nutritional value and bioactive potential. Among plant protein sources studied for BAP production including soybean, pea, rice, corn, and wheat (10, 12, 17), hempseed (*Cannabis sativa* L.) has emerged as a promising alternative. Hempseed protein is nutritionally distinctive among plant proteins, being dominated by the highly digestible storage protein edestin (~60–65% of total protein) and notably enriched in arginine and sulfur-containing amino acids relative to many other plant sources (18, 19). However, compared with these established plant protein sources, hempseed-derived BAPs remain less extensively studied, particularly in relation to processing effects, epithelial passage, and retained bioactivity (16, 20).

### 1.3 Rationale and knowledge gaps for hempseed peptide research

Thermal and non-thermal processing methods followed by GI digestion can substantially influence hempseed protein structure, peptide formation, peptide release, AA composition, and bioactivity (19, 21–23). Therefore, evaluating hempseed-derived peptides after GI digestion and epithelial transport is necessary to determine whether processing-generated bioactivity remains measurable under more physiologically relevant conditions (24, 25). Various *in vitro* antioxidant, anti-inflammatory, and intestinal transport models have been employed to evaluate the biological activity and bioavailability of hempseed-derived peptides (2, 14–16, 26, 27). However, these assays generally measure isolated chemical or cellular responses that do not fully capture physiological digestion, absorption, or systemic bioavailability, underscoring the importance of evaluating hempseed BAPs after simulated GI digestion and epithelial transport, which this review addresses in later sections.

Although undigested hempseed proteins and digests have shown various bioactivities, important knowledge gaps remain. Previous studies of hempseed-derived BAPs have focused on enzyme-treated samples, isolated peptides, peptide identification, or simulated GI digestion as separate outcomes (11, 16, 20, 28, 29). It remains unclear whether non-thermal processing strategies improve the production of peptide fractions with measurable post-digestion and post-transport activity (16, 21, 24). Emerging non-thermal technologies such as ultrasound and high-pressure processing have been shown to alter hempseed protein secondary and tertiary structure, increasing surface hydrophobicity and solubility in ways that may enhance enzyme accessibility and downstream peptide yield (30, 31), though their effect on post-digestion bioactivity specifically remains largely uncharacterized for hempseed. Limited information is also available on whether hempseed peptides remain stable, transportable, and bioactive after simulated GI digestion and epithelial passage. Therefore, this review critically evaluates current evidence regarding the effects of processing and gastrointestinal digestion on hempseed-derived bioactive peptides, with particular emphasis on their antioxidant, anti-inflammatory, antihypertensive, and hypocholesterolemic mechanisms, intestinal bioavailability, and future applications in functional foods and nutraceuticals. This structure–function perspective is applied throughout the review to connect hempseed composition and processing to bioactive outcomes.

The remainder of this review is organized as follows: Section 2 examines hempseed composition as a sustainable plant protein source; Section 3 discusses processing-related generation of bioactive peptides; Section 4 presents mechanistic insights into hempseed BAP-mediated modulation of inflammation; Section 5 addresses gastrointestinal fate, bioaccessibility, and bioavailability; and Section 6 concludes with future research priorities and translational considerations. This is a narrative review; no formal systematic literature search protocol, predefined databases, search strings, inclusion/exclusion criteria, or risk-of-bias assessment were applied. Literature was selected based on thematic relevance to hempseed-derived bioactive peptides, processing, gastrointestinal fate and bioactivity.

## 2 Hemp as sustainable plant-based protein source

Plant-based foods, including cereals, legumes, oilseeds, nuts, fruits, vegetables, and agricultural by-products, are major dietary sources of bioactive compounds (BACs) such as phenolics, flavonoids, carotenoids, vitamins, unsaturated fatty acids, and BAPs (9, 10, 32). These compounds contribute to antioxidant, anti-inflammatory, antidiabetic, anticancer, antimicrobial, and metabolic regulatory activities through mechanisms such as ROS neutralization, lipid oxidation reduction, metal chelation, and modulation of inflammatory pathways, including NF-κB and MAPK signaling (5, 9, 13). Among plant-derived BACs, BAPs are important for functional food and nutraceutical development because plant proteins can serve as renewable precursors for peptides whose activity can be directed by processing, digestion, and fractionation. This makes plant BAPs distinct from many other BACs, as their functional potential depends on both the source protein and the conditions used to generate, preserve, and deliver bioactive peptide sequences (10, 12, 16, 33). Legumes and oilseeds are especially relevant for peptide-related bioactivity since proteins from soybean, pea, lentil, chickpea, rice bran, sesame, flaxseed, and hempseed can release BAPs with reported antioxidant, antihypertensive, anti-inflammatory, antidiabetic, and cholesterol-lowering activities during processing and digestion (10, 12).

Hemp (*Cannabis sativa* L.) is a dicotyledonous plant believed to have originated in Central Asia and one of the earliest domesticated crops, historically used for fiber, seed, oil, textiles, paper, medicinal purposes, and other industrial materials; industrial hemp is now cultivated worldwide as a multipurpose crop for research and commercial applications (18, 34). Hemp also offers agronomic and environmental benefits, including relatively low pesticide requirements, compatibility with organic farming, deep-rooting effects on soil structure, nutrient cycling, and potential support for sustainable cropping systems (34).

Hempseed is nutritionally distinct from many conventional plant protein sources due to its composition within a lipid- and fiber-rich seed matrix (23, 35–39). Compared with soybean, hempseed generally contains lower protein levels but higher oil and fiber contents, giving it a matrix that may influence processing behavior, protein extractability, enzyme accessibility, and subsequent BAP generation (19, 23, 36, 38, 39). As shown in Table 1, hempseed generally contains higher oil content than soybean (23, 38, 39). Hempseed oil is rich in polyunsaturated fatty acids (PUFAs), mainly linoleic acid and α-linolenic acid, with smaller quantities of *γ*-linolenic acid (GLA) that vary by cultivar and growing conditions (18, 19, 23). Hempseed carbohydrate content ranges from 25.49 to 43.0%, mainly due to differences in cultivar, fiber content, structural polysaccharides, and analytical method (23, 37, 39). These carbohydrates are primarily fiber-associated rather than starch-based, with dietary fiber reported at 27.6 g/100 g and predominantly insoluble (18). Most hempseed fiber and minerals are concentrated in the hull, whereas proteins are enriched in the kernel; therefore, dehulling and defatting can increase protein content and digestibility by reducing fiber and anti-nutritional factors (ANFs) such as phytic acid, trypsin inhibitors, and condensed tannins, although removal of hull-rich fractions may reduce mineral content (18, 36, 40). Hempseed protein is considered a complete protein since it provides all nine essential AA with its protein content ranging from 18.3 to 28.92%, compared to 35.6 to 45.3% in soybean (18, 19, 36, 38, 41). The digestibility of hempseed is generally high, ranging from 83% to over 90% depending on cultivar and processing, supporting its classification as a high-quality plant protein (40). Edestin is the predominant storage protein in hempseed, accounting for approximately 60–65% of total protein, followed by albumins (30–35%), and is rich in arginine (Arg) and sulfur-containing AA (SAA: methionine [Met] and cysteine [Cys]) compared with several other plant proteins (19).

**Table 1:** Major constituents of hempseed in comparison to soybean.

Samples	Oil (%)	Carbohydrate (%)	Protein (%)	References
Industrial hempseed varieties	21.12–35.67	25.49–43.0	21.63–28.92	(39)
Industrial hempseed hearts	1.82	—	16.57	(35)
Whole and hulled (Spain)	29.1–32.7	—	18.3–23.0	(36)
Industrial hemp varieties	—	45–57	—	(37)
Commercial hempseed (USA)	31.44	40.78	22.71	(23)
Soybean	18.2–25.4	13.9–35.0	35.6–45.3	(38)

Compared with soybean, lentil, egg, and cow milk, hempseed samples showed different relative levels of AA groups associated with bioactivity (Table 2). Whole hempseed was generally lower than soybean in HBAA (HBAA: alanine, valine, leucine, isoleucine, proline, phenylalanine, and methionine), hydrophilic AAs (HLAA: serine, threonine, asparagine, glutamine, tyrosine, and glycine), branched-chain AAs (BCAA: leucine, isoleucine, and valine), aromatic AAs (AAA: phenylalanine, tyrosine, and tryptophan), positively charged AAs (PCAA: arginine, lysine, and histidine), negatively charged AAs (NCAA: aspartic acid and glutamic acid), and antioxidant AAs (ATAA: tyrosine, tryptophan, phenylalanine, methionine, cysteine, and histidine). However, de-hulled hempseed and hempseed meal contained higher levels of all listed AA groups than egg and cow milk, but lower levels than raw whole lentil seed, indicating that the relative AA-group profile depends strongly on the comparator food and sample form (Table 2) (36, 42–45).

**Table 2:** Amino acid (AA) groups (g/100 g protein) in selected foods.

Samples	HBAA	HLAA	SAA	BCAA	AAA	PCAA	NCAA	ATAA	References
Whole hempseed	6.88	10.07	0.56	3.43	1.94	3.69	6.13	3.46	(36)
Milled hempseed	36.61	52.72	—	17.09	11.07	20.30	21.05	23.68	(23)
Germinated hempseed	42.74	49.07	—	16.39	12.54	17.61	20.75	22.91	(23)
De-hulled hempseed	10.72	15.75	0.94	5.21	3.09	6.78	9.89	5.65	(36)
Hempseed meal	11.41	15.58	0.88	5.71	3.16	6.16	9.69	5.67	(43, 109)
Soybean (U.S.)	24.15	10.52	2.45	12.61	4.35†	16.86	9.17	9.09†	(110)
Lentil, raw whole seed	31.11	16.9	1.94	15.99	9.65	18.71	26.42	14.47	(111)
Egg (whole)	4.06	4.82	0.34	2.51	1.50	2.09	2.92	2.64	(44)
Cow milk	1.43	1.96	0.14	0.83	0.45	0.70	1.37	1.02	(45)

Hydrophobic amino acids (HBAA: alanine, valine, leucine, isoleucine, proline, phenylalanine, and methionine), hydrophilic amino acids (HLAA: serine, threonine, asparagine, glutamine, tyrosine, and glycine), sulfur-containing amino acids (SAA: methionine and cysteine), branched-chain amino acids (BCAA: leucine, isoleucine, and valine), aromatic amino acids (AAA: phenylalanine, tyrosine, and tryptophan), positive charge amino acids (PCAA: arginine, lysine, and histidine), negative charge amino acids (NCAA: aspartic acid and glutamic acid), and antioxidant amino acids (ATAA: tyrosine, tryptophan, phenylalanine, methionine, cysteine, and histidine).

### 2.1 Hempseed as a source of bioactive compounds

Hempseed contains several BACs, including phenolics, tocopherols, phytosterols, and BAPs, which may contribute to its bioactivities (16, 18, 19, 46). Lipid-soluble BACs in hempseed oil include phytosterols and tocopherols, with γ-tocopherol as the predominant tocopherol isomer and total tocopherols commonly ranging from 80 to >150 mg/100 g oil (46, 47). Phenolic compounds are also relevant to hempseed flour as they may contribute to antioxidant activity alongside peptides. Phenolics are generally more concentrated in whole seeds, dehulled seeds, and defatted flours than in oil as many polar antioxidants remain in the seed meal during oil extraction (23, 46). Total phenolic content in hempseed and seed-derived fractions ranges approximately from 0.5 to over 40 mg gallic acid equivalent per gram, with reported compounds including N-trans-caffeoyltyramine, lignanamides, protocatechuic acid, *p*-hydroxybenzoic acid, vanillic acid, *p*-coumaric acid, and ferulic acid (19, 23, 36, 46, 48). Ethanolic extracts of ‘Cheongsam’ hempseeds showed stronger antioxidant and anti-inflammatory activities than stem extracts, along with stronger lipase and α-glucosidase inhibitory activity, attributed to higher levels of phytochemicals such as phenols, condensed tannins, and saponins (49). Although this response reflects differences in plant part and extraction solvent rather than a peptide-mediated processing effect, it illustrates that hempseed’s antioxidant and anti-inflammatory potential is shaped by its overall bioactive compound (BAC) composition, not peptides alone.

Hempseed bioactivity likely reflects contributions from both non-peptide BACs, including phenolics, tocopherols, and phytosterols, and BAPs (11, 50). Among these compounds, BAPs are of particular interest as they can be released from hempseed storage proteins, especially edestin and water-soluble albumin fraction (19, 23, 50). Variation in hempseed peptide bioactivity has been linked to peptide size, AA sequence, AA composition, and degree of hydrolysis (DH), which affect responses across DPPH, TEAC, FRAP, oxygen radical absorbance capacity (ORAC), cellular antioxidant activity (CAA), and intracellular ROS inhibition assays (14, 16, 29, 51). LMW peptides may show stronger bioactivity since their smaller size can improve diffusion, enzyme accessibility, membrane interaction, and binding to receptors or signaling proteins (16, 42, 51). Hempseed-derived peptides and hydrolysates have been reported to suppress inflammatory responses in several cell-based models. For example, hempseed peptides WVSPLAGRT and IGFLIIWV exerted anti-inflammatory activity in LPS-stimulated human hepatic cells by reducing inflammatory responses (20). WVYY and PSLPA activated the Nrf2/HO-1 antioxidant pathway and inhibited NF-κB nuclear translocation in human keratinocytes (52). Rodriguez-Martin et al. (29) further reported that hempseed peptides reduced pro-inflammatory cytokines, including IL-6 and TNF-α, while Khalesi et al. (53) showed that hempseed protein hydrolysates promoted an anti-inflammatory response in human monocytes. Together, these findings indicate that hempseed BAPs may regulate inflammation through combined antioxidant and cytokine-modulating mechanisms, supporting their potential application in functional foods targeting oxidative stress and inflammation.

#### 2.1.1 Influence of amino acids (AA) on bioactivity

Individual AA residues and AA groups influence peptide bioactivity by affecting charge, hydrophobicity, solubility, redox capacity, and target interactions, thereby contributing to antioxidant and anti-inflammatory activities through radical scavenging, electron or hydrogen donation, metal chelation, cellular uptake, membrane interaction, and inflammatory signaling regulation (12, 15, 16, 54, 55). AA composition and residue properties have been linked to antioxidant activity in food-derived peptides, while hempseed-derived peptide studies suggest that peptide sequence and physicochemical properties may also contribute to anti-inflammatory activity (2, 12, 15, 16, 20, 54). However, the presence of these AA residues or AA groups should not be interpreted as evidence that a protein or peptide is inherently beneficial. Peptide effects depend on the complete sequence, concentration, digestive fate, and biological system evaluated (12, 16, 33). Proteins and peptides may also show undesirable properties, including cytotoxicity, allergenicity, pro-oxidant activity, excessive immune stimulation, or other sequence-dependent effects that require separate safety-oriented assays (2, 5, 16). Therefore, AA composition is used here as mechanistic context for interpreting antioxidant and anti-inflammatory screening results, not as confirmation of biological safety or overall health benefit. As with the amino-acid groups discussed throughout this subsection, the following contributions of HBAA and BCAA should be interpreted as one of several modulating factors in peptide bioactivity, not as a standalone predictor (12, 16, 33). HBAA, including BCAA, can improve peptide interactions with lipid environments, oil–water interfaces, membrane bilayers, and membrane-associated signaling targets (2, 54). This hydrophobic affinity may help peptides intercept lipid radicals, reduce lipid peroxidation, enhance membrane permeation, and interact with intracellular pathways such as NF-κB and MAPK, which regulate TNF-α, IL-6, IL-1β, iNOS, and COX-2 expression (2, 5). Udenigwe & Aluko (54) showed that greater hydrophobicity contributed positively to DPPH radical and hydrogen peroxide (H₂O₂) scavenging activity, while Leu contributed strongly to superoxide radical scavenging activity. Beyond their hydrophobic contribution, BCAA may also influence inflammatory responses through metabolic and signaling pathways, including reduced IL-6 and IL-8 production, suppression of NF-κB and MAPK signaling, lower TNF-α and IL-1β levels, M2 macrophage polarization, and autophagy regulation, as well as modulation of JAK–STAT signaling. However, these effects are context-dependent as impaired BCAA catabolism has been associated with oxidative stress and metabolic inflammation in obesity or type 2 diabetes (56). This hydrophobicity-bioactivity relationship is consistent with the hempseed peptide IGFLIIWV (Ile-Gly-Phe-Leu-Ile-Ile-Trp-Val), whose predominantly branched-chain and hydrophobic composition aligns with its reported anti-inflammatory activity in LPS-stimulated hepatic cells (20).

HLAA and charged AAs contribute to peptide bioactivity mainly through solubility, metal binding, redox control, cellular uptake, and interaction with enzymes, receptors, or transcription factors. As with HBAA and BCAA above, the contributions of HLAA and charged residues described below should be interpreted as one of several modulating factors in peptide bioactivity, not as a standalone predictor (12, 16, 33). HLAA, including polar and charged residues such as His, Cys, Glu, Asp, Arg, and Lys, may improve peptide dispersion in aqueous environments, support hydrogen bonding, and increase interaction with polar biological targets (16, 55). His can donate protons and coordinate metal ions through its imidazole ring, while Cys can neutralize radicals through its reactive sulfhydryl group (15, 54). NCAA can support reducing power and chelate transition metals such as Fe^2+^ and Cu^2+^, limiting Fenton-type hydroxyl radical formation (57, 58). Glu may also support glutathione synthesis and cellular redox homeostasis, while Glu and Asp contribute to cellular metabolism and epithelial repair under inflammatory stress (1, 2, 55). PCAA may enhance membrane interaction, cellular uptake, and peptide-target binding, which can support anti-inflammatory responses through suppression of NF-κB and MAPK signaling and reduced cytokine production (2, 5). Supporting this upstream-versus-downstream distinction, Han et al. (4) showed that PCAA-rich oilseed protein hydrolysates reduced TLR4 expression and exhibited direct LPS-binding capacity in murine macrophages, suggesting that positively charged residues may also dampen inflammatory signaling by limiting LPS/TLR4 recognition, in addition to their effects on downstream NF-κB/MAPK pathways. However, the contribution of charged AAs does not show a single consistent pattern across antioxidant assays. Their effects vary with assay mechanism, peptide sequence, neighboring residues, and the measured response. For instance, Udenigwe and Aluko (54) reported that acidic AAs contributed positively to DPPH radical scavenging activity, H₂O₂ scavenging activity, and FRAP, whereas PCAA showed negative contributions in some antioxidant assays, although Lys contributed positively to superoxide radical scavenging activity. The charged-residue contribution described above is reflected in the hempseed peptide WVSPLAGRT (Trp-Val-Ser-Pro-Leu-Ala-Gly-Arg-Thr), which contains both an aromatic (Trp) and a positively charged (Arg) residue and has demonstrated anti-inflammatory activity in hempseed-derived hydrolysates (20).

Consistent with the preceding discussion, the redox and signaling contributions of aromatic and sulfur-containing AAs described below likewise reflect associative trends rather than proof that these residues alone confer bioactivity (12, 16, 33). Aromatic and sulfur-containing AAs provide additional redox and signaling contributions. AAA can stabilize radical intermediates through resonance. Trp has strong electron-donating potential due to its indole ring, Tyr contributes through its phenolic hydroxyl group, and Phe may support hydrophobic interaction and radical stabilization through its aromatic side chain, despite weaker direct antioxidant activity (15, 59). AAA-containing peptides may also contribute to metal chelation when combined with residues such as His and may influence cellular antioxidant defenses through pathways such as nuclear factor erythroid 2-related factor 2/Kelch-like ECH-associated protein 1 (Nrf2/Keap1), which regulates antioxidant enzymes including superoxide dismutase and catalase (57). In addition to their direct redox activity, aromatic AAs may contribute indirectly to inflammatory regulation through metabolic pathways. For example, Trp-derived kynurenine pathway metabolites have been linked to immune regulation and cytokine modulation, while Tyr and Phe may support neuroimmune and metabolic regulation through catecholamine- and thyroid hormone-related pathways (2, 16). However, these effects are context-dependent and should be distinguished from the direct antioxidant activity of aromatic residues within peptide sequences. SAA have been reported to contribute positively to DPPH radical scavenging activity, H₂O₂ scavenging activity, and FRAP, although their effects may differ in superoxide scavenging systems (54). ATAA are relevant since their side chains contain thiol, sulfur-containing, aromatic, imidazole, or basic functional groups involved in redox reactions (15, 54, 59). Thus, although the contribution of AA residues to antioxidant and anti-inflammatory properties depends on the assay and biological system evaluated, processing conditions can be adjusted to enrich peptide fractions with residues associated with targeted redox and inflammatory responses. This is directly exemplified by the hempseed peptides WVYY (Trp-Val-Tyr-Tyr) and PSLPA (Pro-Ser-Leu-Pro-Ala), which contain aromatic (Trp, Tyr) and hydrophobic (Leu, Pro, Ala) residues consistent with the mechanisms above, and which activated Nrf2/HO-1 signaling and inhibited NF-κB nuclear translocation in human keratinocytes (52).

#### 2.1.2 Influence of peptide concentration on antioxidant and pro-oxidant activity

Although BAP-mediated antioxidant activity is generally considered beneficial, these effects are not uniformly protective across all concentrations. Redox-active compounds, including small-molecule antioxidants, can display a biphasic, concentration-dependent relationship in which antioxidant behavior at low doses shifts toward pro-oxidant activity at higher concentrations. For example, Trolox, the reference standard used in the TEAC assay, exhibits antioxidant activity at low micromolar concentrations but switches to pro-oxidant behavior at higher doses in cellular models (60). This pattern reflects a hormetic response, in which low-dose exposure activates adaptive cellular defense pathways such as Nrf2/Keap1, whereas excessive concentrations overwhelm these defenses and instead promote ROS generation and oxidative damage (61, 62). Comparable concentration-dependent switching has been reported for other redox mediators; for instance, hydrogen peroxide enhances insulin signaling at low concentrations but impairs it at higher doses (63). Although this concentration-dependent phenomenon has been characterized primarily in small-molecule antioxidants and redox mediators rather than in peptides specifically, the underlying mechanisms; electron donation, metal chelation, and cellular redox threshold effects are chemically relevant to peptide-based antioxidants as well, and comparable dual behavior cannot be excluded for hempseed-derived peptides at high concentrations. From a nutraceutical development perspective, establishing a safe and effective concentration range, rather than assuming that greater peptide dose or measured antioxidant capacity is uniformly beneficial, will be necessary before hempseed-derived BAPs can be advanced into functional food or supplement applications (2, 5, 16). Until definitive hempseed-peptide dose–response data are available, nutraceutical formulation should avoid assuming monotonic beneficial effects with increasing peptide concentration.

#### 2.1.3 Impact of processing on hempseed composition and bioactivity

Thermal and non-thermal processing methods (Figure 3) can alter hempseed matrix structure and composition, thereby affecting protein accessibility, peptide generation, digestibility, bioactivity, and bioavailability of hempseed-derived ingredients (18, 42) (Table 3).

**Figure 3:**
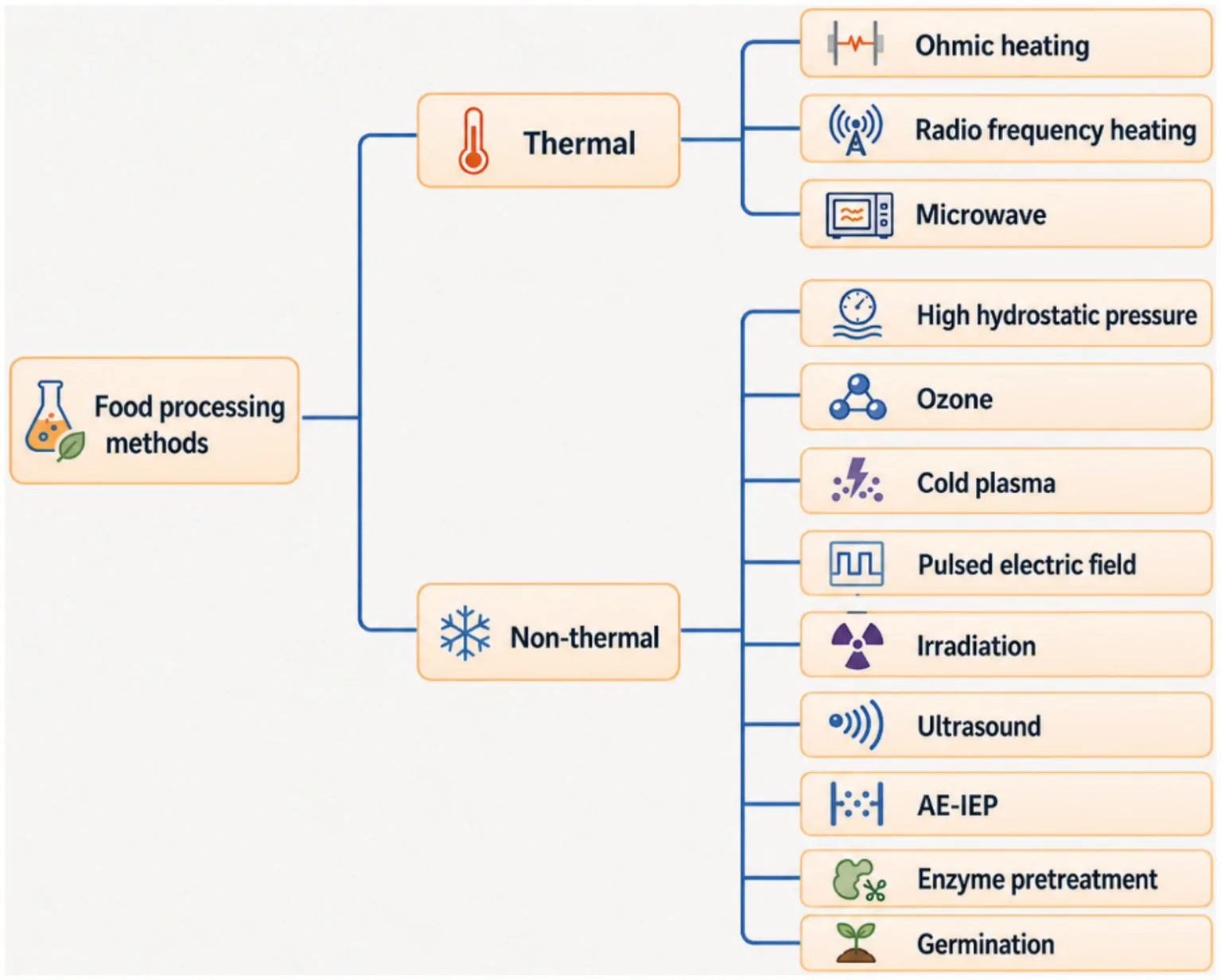
Thermal and non-thermal food processing methods.

**Table 3:** Effects of thermal and non-thermal methods of processing on BAPs.

Parameter	Processing methods		References
	Thermal	Non-thermal	
Structural stability	Heating above 80 °C denatures hempseed protein isolate (denaturation temperature ~92 °C), reduces solubility, and promotes edestin aggregation driven by disulfide bond formation and a shift toward intermolecular *β*-sheet structure, reducing enzyme accessibility	Ultrasound-assisted extraction (20 kHz) improves hempseed protein isolate solubility ~2-fold via cavitation-induced structural changes, increasing surface hydrophobicity and free thiol exposure without the same aggregation-driven insolubility seen with heat	Thermal: (65–67) Non-thermal: (30)
Bioactivity impact	Heat pretreatment (100 °C, 15–30 min) prior to pepsin-pancreatin digestion improved hempseed protein digestibility (increased LMW peptide yield, degree of hydrolysis) but reduced antioxidant, anti-proliferative, and anti-inflammatory activity of the resulting digests relative to unheated protein. Roasting alone increased phenolic content (~155%) and antioxidant activity in whole hempseed	Enzymatic hydrolysis (Alcalase, Flavourzyme, bromelain) of hempseed protein has generated peptides with confirmed anti-inflammatory (TLR4/MD2 docking), antihypertensive (ACE-inhibitory), and hypocholesterolemic activity, directly measured in cell and human models	Thermal: (86, 91, 92, 112, 113)
Industrial scalability	Well-established, low-cost equipment (boiling, roasting, extrusion) at industrial scale, but energy-intensive and associated with nutrient/BAC loss	Ultrasound and HPP show promising protein recovery (~62%) and purity (~76%) at pilot scale, but remain less established industrially for hempseed specifically, with higher capital equipment costs	(31)

##### 2.1.3.1 Thermal processing

Thermal processing, including boiling, roasting, autoclaving, high-pressure cooking, and extrusion, commonly exposes food materials to elevated temperatures to inactivate microorganisms and endogenous enzymes, improve safety, and modify food structure (42). In hempseed specifically, differential scanning calorimetry has shown a protein denaturation temperature of ~92 °C, with heating above 80 °C significantly reducing hempseed protein isolate solubility and promoting large protein aggregate formation (64). At the structural level, heat disrupts the edestin hexamer characteristic of hempseed’s dominant storage protein, with thermal aggregation of edestin primarily driven by disulfide bond formation; notably, heating has also been shown to increase the proteolysis rate of edestin’s acidic and basic subunits, directly supporting the mechanism by which moderate thermal treatment can enhance downstream peptide release (65). This structural unfolding is consistent with the mechanism by which moderate thermal treatment (63–72 °C) can promote controlled protein unfolding without severe aggregation, thereby increasing protein flexibility and peptide liberation. However, more extensive thermal denaturation shifts hempseed protein secondary structure toward intermolecular *β*-sheet formation, stabilized by hydrophobic and disulfide linkages, resulting in insoluble aggregates with reduced enzyme accessibility (66). Severe heating can also induce Maillard reactions, alter AA residues, reduce digestibility, and decrease peptide bioactivity, while heat-induced hempseed protein aggregation has additionally been linked to altered oxidative stability of the resulting digested peptides (67). In addition, excessive heat can degrade heat-sensitive nutrients and BACs, including some vitamins, PUFAs, and phenolic antioxidants (18).

##### 2.1.3.2 Non-thermal processing

Non-thermal approaches such as germination, fermentation, mechanical size reduction, fractionation, dehulling, pH-shift extraction and enzyme pretreatment can modify seed matrices while limiting heat-induced damage to sensitive nutrients and bioactive compounds (23, 42, 43, 68). Under milder conditions compared to conventional thermal processing, these methods can promote controlled structural modification, improve protein accessibility, enhance the release or extractability of peptides and BACs, and reduce ANFs, all without extensive heat exposure (19, 23, 48).

##### 2.1.3.3 Germination

Germination is a simple, cost-effective non-thermal process used to improve the functional properties of seeds and pulses. It involves water uptake, activation of endogenous enzymes, mobilization of storage reserves, and radicle emergence (69, 70). During germination, proteases, amylases, phytases, and cell-wall-degrading enzymes modify storage proteins, carbohydrates, phytate, and cell-wall components, thereby increasing the accessibility of BACs such as peptides, free AAs, tocopherols, vitamin C, and other polyphenols (19, 21, 48). The extent of these changes depends on genotype, seed vigor, maturity, dormancy, temperature, moisture, oxygen, light, and germination time (69, 70). In hempseed, germination can alter protein, phenolic, and ANF profiles by promoting reserve mobilization and partial hydrolysis of storage proteins. These changes may modify the AA profile available for subsequent hydrolysis or digestion and improve the release of antioxidant-active compounds. Germination has been reported to increase total phenolic content (TPC), total flavonoid content (TFC), and antioxidant capacity in hempseed compared with raw seeds, while maintaining relatively stable PUFA profiles (36, 69). The increases in phenolic content and antioxidant activity are mainly associated with greater accessibility of antioxidant compounds and formation of smaller bioactive fragments during germination and subsequent enzymatic treatment or digestion (48, 69, 70). Evidence from other plant protein sources supports this mechanism. In lentils, germination improved protein digestibility, antioxidant activity, and ACE inhibition, especially in LMW peptide fractions <3 kDa (71). Similarly, germinated soybean hydrolysates showed stronger antioxidant activity than non-germinated controls, likely due to biochemical changes that increased phenolic availability and promoted smaller peptide formation (17, 70). Collectively, these findings indicate that germination may prepare hempseed proteins and non-peptide BACs for stronger responses during subsequent enzyme treatment or GI digestion. Germination may also influence anti-inflammatory properties, although this effect remains less studied in hempseed than antioxidant activity. Protease activation can increase the availability of peptides and free AAs that may participate in inflammatory regulation, while increased phenolic availability may indirectly reduce inflammation by lowering oxidative stress linked to inflammatory signaling (36, 48). Evidence from related legume systems indicates that germination can promote BAP formation with anti-inflammatory potential, supporting its relevance as a processing strategy for improving hempseed bioactivity (11).

###### 2.1.3.3.1 Fermentation

Fermentation is a non-thermal approach that uses microorganisms, including bacteria, yeasts, and filamentous fungi, to modify the nutritional, functional, and sensory properties of foods (9). During fermentation, microbial enzymes such as proteases, amylases, lipases, esterases, glycosidases, and cell-wall-degrading enzymes break down macromolecules, reduce ANFs, improve nutrient bioavailability, and generate flavor and aroma compounds (43, 48). In hempseed products specifically, fermentation directly modifies protein structure and promotes peptide release. Solid-state fermentation of hempseed cake with the fungus *Aspergillus niger*, compared against the bacteria *Bacillus subtilis* and *Lactobacillus rhamnosus*, significantly increased degree of hydrolysis, soluble protein content, and free amino acid content across all three microorganisms, with *B. subtilis* producing the largest increases (degree of hydrolysis from 2.77 to 48.72%; soluble protein from 28.12 to 208.21 mg/g; free amino acids from 4.09 to 52.81 mg/g), and SDS-PAGE directly confirming the breakdown of macromolecular hempseed proteins into peptides (72). Similarly, solid-state fermentation of hemp-pressed cake with the fungus *Rhizopus oligosporus* preserved total protein content while progressively releasing free amino acids over the fermentation period (73). Bioprocessing of hemp screenings using yeast, enzyme mixtures, and combinations of both has similarly been shown to increase nutrient and phytochemical bioavailability during simulated digestion and microbial fermentation (74). Beyond protein-focused outcomes, fermentation with lactic acid bacteria has also been shown to modify hempseed’s non-peptide bioactive compounds: fermentation of hemp bran with *Lactiplantibacillus plantarum*, *Limosilactobacillus fermentum*, and *Lacticaseibacillus rhamnosus* generated new terpenes and increased short-chain fatty acids, including acetic, propionic, and butyric acids (75), and Lactobacillus-fermented hempseed preparations showed increased phenolic content, flavonoid content, and antioxidant activity (76). However, the magnitude and direction of fermentation effects on both protein and non-protein hempseed constituents depend on microbial strain, fermentation duration, substrate composition, and extraction method (48, 77).

###### 2.1.3.3.2 Protein fractionation

Protein fractionation is widely used to convert hempseed flour or defatted meal into protein-rich ingredients (23, 25). Wet or dry fractionation can produce protein-, fiber-, and lipid-associated fractions, while alkaline extraction-isoelectric precipitation (AE-IEP) is commonly used to recover hempseed protein concentrates or isolates with high purity, often exceeding 85–90% depending on extraction conditions (23, 25, 43). In AE-IEP, defatted hempseed flour is solubilized under alkaline conditions, commonly pH 8–10, followed by protein precipitation near pH 4.5–5.0. This process relies on pH-dependent protein solubility: alkaline pH increases protein-water interactions and electrostatic repulsion, allowing protein separation from the seed matrix, whereas the isoelectric point reduces net charge and promotes aggregation and protein recovery (25, 78). Alkaline extraction-isoelectric precipitation (AE-IEP) substantially modifies hempseed composition and biofunctional potential by enriching storage proteins such as edestin and water-soluble albumin fraction while reducing fiber, carbohydrates, lipids, phenolics, minerals, and some ANFs (19, 21, 25, 43). This creates a protein-concentrated substrate with greater enzyme accessibility for subsequent hydrolysis or simulated GI digestion (19, 21, 25, 43). Enzyme-treated products from alkali-extracted hemp protein isolates have shown stronger antioxidant and ACE-inhibitory activities than less refined protein systems, suggesting that protein enrichment can increase peptide precursor availability during controlled hydrolysis (79). However, protein isolates may not always show higher antioxidant capacity before hydrolysis as removal of phenolic co-factors and other BACs can reduce non-peptide antioxidant contributions (22, 23, 43). Although AE-IEP improves protein concentration, processing variables such as pH, extraction time, temperature, and precipitation conditions can affect protein solubility, digestibility, enzyme accessibility, and downstream functionality (22, 25, 43). Harsh alkaline or acid exposure may partially denature proteins, promote aggregation, reduce solubility or digestibility, alter susceptible AAs such as Lys, Cys, Met, Ser, Thr, and Trp, and produce undesirable reaction products (22, 43). These changes may affect antioxidant and anti-inflammatory potential by altering protein purity, matrix structure, retention of minor BACs, and the peptide fractions generated during downstream enzyme treatment or GI digestion (22, 25).

Related approaches can further support protein recovery or functionality. pH-shift processing also exposes proteins to extreme pH followed by neutralization, and is often used to modify techno-functional properties such as solubility, dispersion, emulsion stability, and oxidative resistance rather than for protein separation alone (80). Mechanical size reduction, including milling or grinding, can improve extraction and hydrolysis by disrupting cell-wall structures, increasing surface area, and exposing protein bodies and intracellular components (43). After enzyme treatment or digestion, membrane fractionation can enrich peptide fractions by MW. In hempseed hydrolysates, low-MW fractions, often <10 kDa, have shown stronger radical-scavenging activity than unfractionated hydrolysates, although metal-chelating capacity may vary with MW range and DH (36, 81). Nanofiltration and ultrafiltration are also used industrially to recover BAP-rich fractions, while mass spectrometry supports peptide identification and sequencing in hempseed and other proteins (11, 16, 25, 78).

###### 2.1.3.3.3 Enzyme hydrolysis

Enzyme pretreatment is one of the most direct and efficient non-thermal processing strategies for generating BAPs from hempseed proteins (Table 4) (16, 19, 23, 41). Enzyme pretreatment may also enhance anti-inflammatory and broader health-related properties by releasing peptide sequences capable of interacting with inflammatory and metabolic pathways (10, 11, 53). Hempseed peptide fractions generated by enzymatic hydrolysis or simulated GI digestion have shown anti-inflammatory, antiproliferative, cholesterol-lowering, immunomodulatory, antihypertensive, neuroprotective, anti-neuroinflammatory, and Nrf2-associated cytoprotective effects (10, 11, 42, 52, 81, 82). Proteases cleave peptide bonds under mild aqueous conditions, commonly pH 6–9 and temperatures ≤60 °C, producing oligopeptides and free AAs while preserving heat-sensitive compounds and minimizing Maillard-type crosslink formation (19, 23, 42, 80, 83). Endopeptidases such as Alcalase and Neutrase cleave internal peptide bonds, whereas exopeptidases such as Flavourzyme release AA or short peptides from the peptide termini. Other protease and enzymes, including bromelain, pepsin, pancreatin, and multi-enzyme cocktails, have also been used to generate hempseed hydrolysates (41, 84). Enzyme specificity, substrate structure, hydrolysis conditions, and other processing methods determine DH, MW profile, AA composition, hydrophobicity, and sequence characteristics, which together shape the bioactive properties of hempseed-derived peptides (16, 42, 84). Consistent with this, Zhang et al. (41) showed that simulated GI digestion of hemp seed protein generated a complex peptide profile and improved antioxidant activity. In hempseed, peptide release can be affected by the edestin/albumin ratio, disulfide pattern, protein conformation, aggregation behavior, and surrounding matrix components (25, 42, 43). Flour-based substrates contain proteins together with fiber, lipids, phenolics, and other matrix components that can dilute protein concentration or interact with enzymes, whereas protein isolates provide a more concentrated protein substrate but may undergo structural changes during extraction or precipitation. These differences help explain variation in DH, peptide release, and bioactivity among hempseed flours, isolates, hydrolysates, and digests (25, 42, 43, 83, 85).

**Table 4:** Bioactivity of enzyme-pretreated hempseed peptides.

Bioactivity	Enzyme	Summary	References
Antioxidant and ACA inhibitory	Alcalase	Alcalase hydrolysis of hemp bran protein generated peptide fractions with radical-scavenging, metal-binding, ferric-reducing, and ACE-inhibitory activities.	(82)
	Alcalase and Flavourzyme	Hemp protein hydrolysates produced with Alcalase and Flavourzyme showed DPPH radical-scavenging activity, *β*-carotene bleaching inhibition, and FRAP; shorter peptide sequences were associated with stronger radical-scavenging response.	(50)
Antihypertensive	Pepsin followed by pancreatin	Hempseed protein and hempseed protein plus hydrolysate-derived peptides reduced 24-h systolic and diastolic blood pressure in adults with mild hypertension compared with casein.	(95)
Anti-inflammatory	Alcalase and bromelain	Hempseed protein hydrolysates reduced inflammatory mediators in LPS-stimulated macrophages; Alcalase hydrolysate showed stronger inhibition of NO, IL-6, and TNF-α than bromelain hydrolysate.	(112)
Immunomodulatory/anti-inflammatory	Alcalase followed by Flavourzyme	Bioavailable hempseed protein hydrolysates obtained after Caco-2 transport showed immunomodulatory activity; six oligopeptides were identified and experimentally validated in primary human monocytes, with molecular docking suggesting interaction with TLR4/MD2	(92)
Anti-inflammatory	Alcalase and Flavourzyme	HPHs decreased the pro-inflammatory mediators (TNF-α, IL-1β, and IL-6) while increasing anti-inflammatory mediators (IL-10 and IL-4)	(29)
Cholesterol-lowering	Pepsin	Hempseed peptide H3, IGFLIIWV, obtained from pepsin-hydrolyzed hempseed protein, exerted cholesterol-lowering effects in human HepG2 hepatic cells	(96)

Controlled hydrolysis can shift hempseed proteins toward low- and medium-MW peptide fractions with greater exposure of functional residues (10, 16, 42). However, excessive cleavage may disrupt active sequences or generate fragments and free AAs with lower activity (12, 85). Hempseed peptide fractions may show stronger antioxidant activity than parent proteins as enzyme treatment exposes or releases LMW peptides containing functional residues that support lipid-radical interaction, electron or hydrogen donation, metal chelation, and aqueous solubility (10, 14, 42). Across hempseed and related proteins, enzymatic hydrolysis has enhanced DPPH radical scavenging activity, TEAC, ORAC, FRAP, ACE-inhibitory, and cellular antioxidant activities (10, 79, 82). These responses are generally linked to LMW peptides, hydrophobic and aromatic residues, and peptide interactions with target enzymes or signaling pathways through hydrophobic, hydrogen-bonding, or electrostatic interactions (10, 16, 42). Overall, enzyme pretreatment converts hempseed storage proteins into peptide fractions with distinct physicochemical and bioactive properties, but post-treatment effectiveness must be interpreted together with downstream digestion and bioavailability.

Thermal and non-thermal processing methods influence hempseed BAP structure and bioactivity through distinct mechanisms (Table 3). Thermal treatment improves protein digestibility by promoting structural unfolding and increasing enzyme accessibility, but this benefit is temperature-dependent: moderate heat (63–72 °C) enhances peptide liberation, while heat above 80–92 °C denatures hempseed protein and drives edestin aggregation via disulfide bond formation, ultimately reducing enzyme accessibility and, as demonstrated in the study by Lin et al. (86) yielding digests with lower antioxidant and anti-inflammatory activity than unheated controls. Non-thermal approaches, particularly enzymatic hydrolysis, avoid this trade-off by generating bioactive peptides directly without inducing the same degree of protein aggregation, and have produced hempseed peptides with confirmed antioxidant, anti-inflammatory, antihypertensive, and hypocholesterolemic activity across cell and human intervention studies. Emerging non-thermal technologies (ultrasound, microwave, high-pressure processing) further improve protein solubility and extraction yield without the aggregation-driven losses associated with high-temperature treatment, though industrial-scale data for hempseed specifically remain more limited than for conventional thermal methods. Collectively, these findings suggest that non-thermal and hybrid processing strategies may offer advantages for preserving hempseed BAP bioactivity, while thermal processing remains advantageous primarily for digestibility enhancement and microbial safety.

###### 2.1.3.3.4 Novel processing methods

Emerging non-thermal technologies, including ultrasound-, microwave-, and high-pressure-assisted extraction, have also been applied to hempseed protein recovery. High-intensity ultrasound-assisted extraction (20 kHz) improved hempseed protein isolate solubility approximately 2-fold relative to conventional alkaline extraction-isoelectric precipitation, attributed to acoustic cavitation-induced changes in secondary and tertiary protein structure, increased surface hydrophobicity, and greater exposure of free thiol groups (30). Microwave-assisted subcritical water extraction has similarly been used to simultaneously recover protein and phenolic compounds from hempseed (87). Combining high-pressure processing (HPP, 200 MPa) with ultrasound-assisted extraction on defatted hempseed cake achieved protein recovery of approximately 62% and purity of approximately 76%, with ultrasound-assisted extraction independently improving amino acid extraction yield relative to conventional methods (31). While these studies have primarily characterized extraction yield and protein structural changes rather than downstream bioactive peptide generation directly, the structural modifications reported (increased surface hydrophobicity, altered disulfide/thiol chemistry) are consistent with the processing-bioactivity relationships discussed elsewhere in this section, indicating that these emerging technologies represent a promising but still underexplored avenue for hempseed BAP production.

###### 2.1.3.3.5 Hybrid processing strategies for hempseed peptide yield

Combining or directly comparing thermal and non-thermal processing strategies has emerged as a promising approach for optimizing hempseed peptide yield. Wang et al. (88) directly compared heating (90 °C, 30 min) against ultrasound (200, 400, and 600 W) as pretreatments prior to enzymatic hydrolysis of hempseed protein isolate, finding that moderate-power ultrasound (200 W) outperformed both heating and higher-power ultrasound, yielding a greater degree of hydrolysis, a higher proportion of low-molecular-weight peptides (1.0–3.0 kDa), and stronger antioxidant and ACE-inhibitory activity, corresponding to greater protein unfolding, increased surface hydrophobicity, and structural changes confirmed by SDS-PAGE (88). Beyond thermal-versus-non-thermal comparisons, combining two non-thermal techniques has also shown clear synergistic effects for hempseed protein: sequential high-pressure homogenization and high-intensity ultrasound produced a synergistic increase in hempseed protein isolate solubility (from 6.88 to 22.89% at neutral pH), substantially exceeding either treatment alone, alongside reduced particle size and *β*-sheet content and increased surface hydrophobicity (89). Similarly, combining solid-state fermentation and germination with high-intensity ultrasound has been shown to degrade hempseed protein into nano-sized peptides, illustrating that hybrid non-thermal strategies can directly enhance peptide-scale outcomes (90). Collectively, these findings indicate that hybrid processing, whether contrasting thermal and non-thermal pretreatments or combining multiple non-thermal techniques, can synergistically improve hempseed protein unfolding, peptide yield, and downstream bioactivity beyond what single treatments achieve alone. It should be noted that all hybrid-processing data reported to date for hempseed derive from pilot- or laboratory-scale studies; industrial-scale demonstration of these combined techniques for hempseed remains scarce, consistent with the broader technical scale-up challenges discussed in Section 2.6.

### 2.2 Hydrolyzed proteins and bioactive peptide in inflammation: mechanistic insights

Figure 4 illustrates the proposed mechanisms by which hydrolyzed, and plant-derived BAPs regulate inflammation in a hypothetical eukaryotic cell. White adipose tissue (WAT) functions not only as an energy-storage tissue but also as an endocrine and immune organ that communicates with the central nervous system and regulates metabolic homeostasis through adipokine and cytokine secretion (91). During excess fat accumulation, WAT undergoes metabolic stress, promotes M1-like macrophage infiltration, and increases IL-1β, TNF-α, and IL-6 production, contributing to chronic low-grade inflammation, impaired energy expenditure, and metabolic dysfunction (3). IL-1β can further disrupt adipocyte mitochondrial integrity and suppress brown adipose tissue (BAT) thermogenic activity, worsening lipid accumulation (91). A key pathway involved in this response is the LPS/LBP-TLR4-MyD88-NF-κB axis. LPS and IL-1β activate TLR4 and IL-1R, respectively, leading to MyD88 recruitment, IκBα phosphorylation and degradation, NF-κB nuclear translocation, and increased transcription of inflammatory mediators, including COX-2, iNOS, MCP-1, MIP-1, and IP-10 (1). NF-κB signaling also intersects with the JAK–STAT pathway, as pro-inflammatory cytokines such as IL-6 and TNF-α can activate JAK kinases and downstream STAT3, which cooperates with NF-κB to sustain transcription of inflammatory mediators and adipokines, while suppressor of cytokine signaling (SOCS) proteins act as negative-feedback regulators that limit this crosstalk and constrain prolonged macrophage activation. This NF-κB/JAK–STAT/SOCS crosstalk is therefore a relevant target for hempseed-derived BAPs specifically, as the hempseed peptides discussed below (WVSPLAGRT, IGFLIIWV, WVYY, PSLPA) act on overlapping NF-κB and Nrf2/HO-1 nodes within this same signaling network (1, 53).

**Figure 4:**
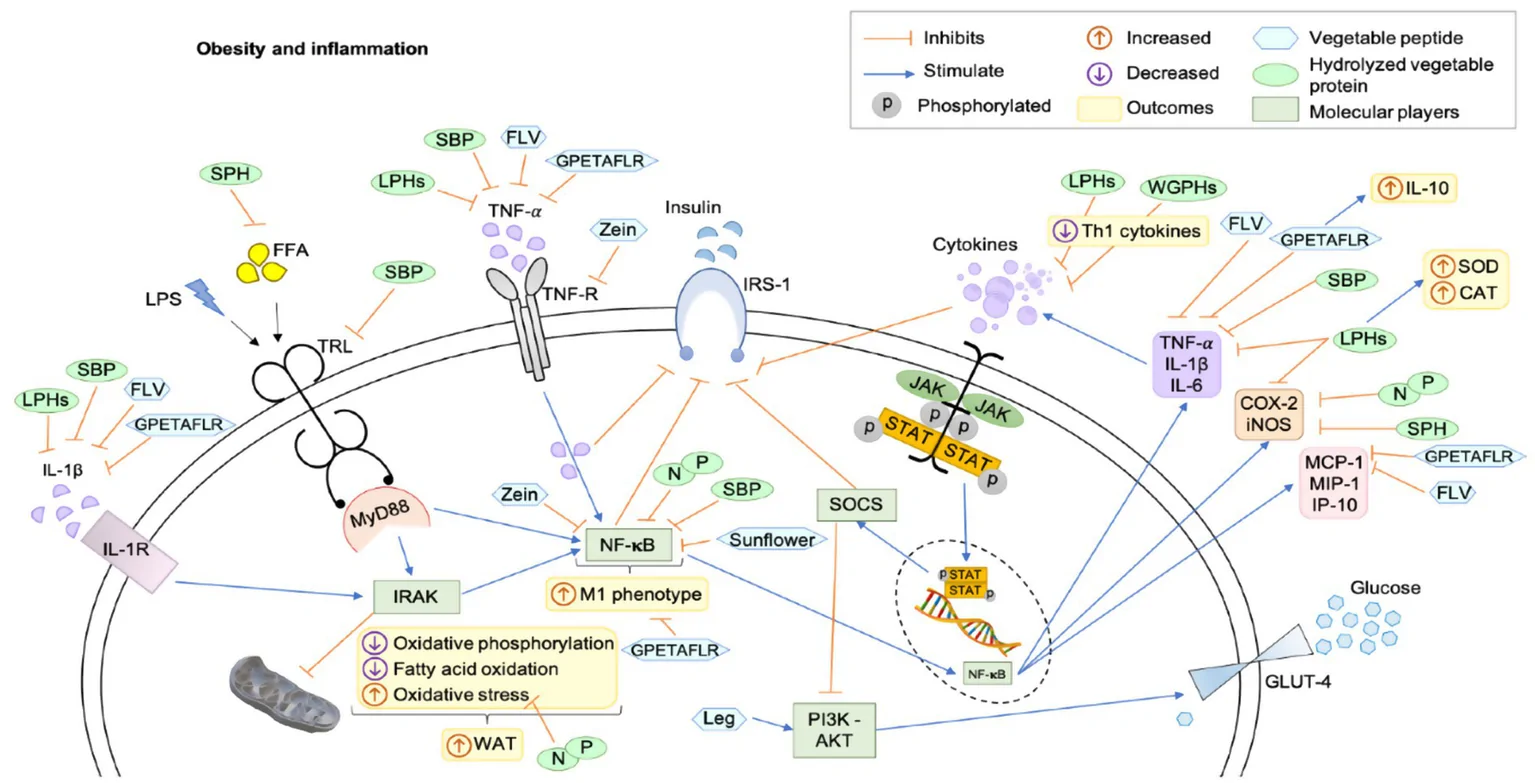
Proposed anti-inflammatory mechanisms of hydrolyzed proteins and plant-derived peptides on obesity and inflammation in a hypothetical eukaryotic cell. Adapted from de Medeiros et al. (91), licensed under CC BY 4.0. Hydrolyzed proteins and peptides reduced inflammation via metabolic pathways responsible for positive feedback in adipose tissue accumulation and subclinical inflammation, with some compounds aiding detoxification and reducing oxidative stress. CAT, catalase; COX-2, cyclooxygenase-2; FFA, free fatty acid; FLV, soy peptide Phe-Leu-Val; GLUT4, glucose transporter type 4; GPETAFLR, peptide from *Lupinus angustifolius* L., IL-10, interleukins-10; IL-6, interleukins-6; IL1-β, interleukins-1β; IL1-R, interleukins-1-receptor; iNOS, inducible nitric oxide synthase, IP-10, interferon-inducible protein 10; IRAK, IL-1R-associated kinase 2; IRS-1, insulin receptor substrate 1; JAK–STAT, Janus kinase-signal transducer and activator of transcription, Leg, leginsulin; LPHs, lupine protein hydrolysates; LPS, lipopolysaccharides; M1, M1-like macrophage; MCP-1, monocyte chemoattractant protein-1; MIP-1, macrophage inflammatory protein-1; MyD88, myeloid differentiation primary response 88; N and P, protein hydrolysates of the common bean (*Phaseolus vulgaris* L.) varieties Negro 8025 and Pinto Durango; NFκB, nuclear factor kappa B; PI3K-AKT, phosphatidylinositol 3-kinase/protein kinase B; SBP, soybean protein-derived peptides; SOCS, suppressor of cytokine signaling; SOD, superoxide dismutase, SPH, soy protein peptic hydrolysate; Sunflower, peptides from sunflower protein hydrolysate; Th1, T helper 1, TNF-α, tumor necrosis factor-α; TNF-R, tumor necrosis factor-receptor; TLR, toll-like receptor; WAT, white adipose tissue; WGPHs, wheat gluten protein hydrolysates; Zein, peptides for zein hydrolysate.

Published molecular docking data specific to hempseed-derived peptides support this receptor-level mechanism. Millan-Linares et al. (92) identified six bioavailable hempseed-derived oligopeptides (DDNPRRF, SRRFHLA, RNIFKGF, VREPVFSF, QADIFNPR, and SAERGFLY), obtained following simulated Caco-2 transepithelial transport, and characterized their molecular docking interactions with the TLR4/MD2 receptor complex at the residue level. These peptides subsequently reduced pro-inflammatory cytokine gene expression (IL-1β, IL-6, TNF-α) and modulated IL-4, IL-10, and TLR4 expression in primary human monocytes, directly linking structural docking predictions to functional anti-inflammatory outcomes. This hempseed-specific structural evidence supports peptide-TLR4 interaction as a plausible upstream entry point into the LPS/TLR4/MyD88/NF-κB axis described above. Direct docking of hempseed-derived peptides against NF-κB itself has not yet been reported in the literature; as NF-κB acts intracellularly downstream of TLR4/MyD88 activation, receptor-level docking at TLR4/MD2 represents the most direct structural evidence currently available for this pathway.

Anti-inflammatory BAPs generally suppress inflammatory activation by reducing NO, IL-6, TNF-α, IL-1β, iNOS, and COX-2 production and by modulating NF-κB, MAPK, and Nrf2/HO-1 signaling pathways (1, 2, 5). This pattern is directly reflected in hempseed-derived peptides: WVSPLAGRT and IGFLIIWV reduced inflammatory responses in LPS-stimulated human hepatic cells, WVYY and PSLPA activated Nrf2/HO-1 and inhibited NF-κB nuclear translocation in human keratinocytes, and hempseed protein hydrolysates reduced IL-6 and TNF-α while promoting anti-inflammatory responses in human monocytes (20, 29, 57, 58). Comparable NF-κB- and MAPK-modulating activity has also been reported for peptides from other plant sources (e.g., soy, lupine, wheat gluten), indicating that this mechanism is conserved across plant-protein-derived BAPs rather than unique to a single source (4, 53).

LPS, a structural component of gram-negative bacterial outer membranes, is widely used to induce inflammation in RAW 264.7 macrophages. LPS stimulation increases TNF-α, IL-1β, IL-6, NO, and PGE2 production through iNOS and COX-2 activation (9, 93). These responses are mainly regulated by NF-κB and MAPK signaling, including ERK, JNK, and p38 phosphorylation. Gut microbiota dysbiosis can also increase circulating LPS, which activates intestinal epithelial and immune cells and contributes to persistent inflammatory and metabolic disorders (9, 93). This information supports the use of LPS-stimulated RAW 264.7 macrophages as a screening model for evaluating the anti-inflammatory potential of hempseed-derived peptides specifically, as applied throughout the hempseed-specific evidence presented in this section.

### 2.3 Mechanistic insights into antihypertensive and hypocholesterolemic activities

Beyond antioxidant and anti-inflammatory pathways, hempseed-derived peptides have demonstrated mechanistic activity against hypertension and dyslipidemia, two conditions closely linked to oxidative stress and chronic inflammation. Antihypertensive activity is primarily mediated through inhibition of angiotensin-converting enzyme (ACE), a key regulator of the renin-angiotensin system that converts angiotensin I into the vasoconstrictor angiotensin II and inactivates the vasodilator bradykinin, most commonly via competitive inhibition at the ACE active site (94). Samaei et al. (82) identified 35 ACE-inhibitory peptides from Alcalase-hydrolyzed hemp bran protein fractions using high-resolution mass spectrometry, with structural features (low molecular weight, specific C-terminal residues) correlating with ACE-inhibitory potency, alongside concurrent antioxidant activity in the same hydrolysate. These *in vitro* findings are supported by translational evidence from Samsamikor et al. (95), who reported that hemp seed protein and its hydrolysate lowered blood pressure in a double-blind crossover trial in adults with hypertension, compared with casein protein, indicating that ACE-inhibitory activity observed *in vitro* may translate to measurable antihypertensive effects in humans.

Hypocholesterolemic activity has been mechanistically characterized for the hempseed-derived peptide H3 (IGFLIIWV) by Li et al. (96), who showed that H3 inhibits 3-hydroxy-3-methylglutaryl coenzyme A reductase (HMG-CoAR), the rate-limiting enzyme in cholesterol biosynthesis, in a dose-dependent manner. H3 additionally activated the sterol regulatory element-binding protein-2 (SREBP-2) transcription factor, increasing low-density lipoprotein receptor (LDLR) expression and enhancing cellular LDL uptake in human hepatic (HepG2) cells. H3 further increased HMG-CoAR phosphorylation via AMP-activated protein kinase (AMPK) activation, and reduced proprotein convertase subtilisin/kexin type 9 (PCSK9) secretion through decreased hepatic nuclear factor 1-alpha (HNF1-α) expression, collectively enhancing hepatic cholesterol clearance through a mechanism resembling that of statin therapy. Together, these hempseed-specific findings indicate that antihypertensive and hypocholesterolemic bioactivities, like antioxidant and anti-inflammatory effects, are mediated through defined molecular targets (ACE, HMG-CoAR, SREBP-2/LDLR, PCSK9) rather than general peptide bulk properties, supporting a structure–function framework consistent with the mechanistic approach applied Section 2.2.

### 2.4 Fate of proteins/peptides along the gastrointestinal tract (GIT)

BAPs may occur naturally in foods or remain encrypted within precursor proteins until released by enzyme pretreatment, fermentation, or GI digestion (16, 51). During GI digestion, food proteins and released peptides are exposed to multiple enzymes, including pepsin in the stomach; trypsin, chymotrypsin, elastase, and carboxypeptidases in the small intestine; and brush-border peptidases on the intestinal epithelium. These enzymes can modify peptide structure, stability, and bioactivity (16, 46, 81). During the gastric phase, acidic pH activates pepsin, which preferentially cleaves peptide bonds near HBAA such as Phe and Leu. This process generates shorter fragments that may retain, increase, or lose bioactivity depending on their sequence and stability (46, 81). In the intestinal phase, near-neutral to slightly alkaline pH activates pancreatic enzymes. Trypsin cleaves after basic residues such as Arg and Lys, chymotrypsin targets aromatic residues such as Phe, Tyr, and Trp, and elastase cleaves near small neutral residues such as Ala, Gly, and Ser. Carboxypeptidases and brush-border enzymes, including aminopeptidase N and aminopeptidase A, further hydrolyze oligopeptides into smaller peptides and free AAs (16, 46, 76, 81).

GI digestion is therefore not only degradative; it can release new bioactive sequences, transform existing peptides, or degrade BAPs into fragments with lower or no bioactivity (16, 51). This concept is supported by Zhang et al. (41), who showed that simulated GI digestion of hemp seed protein generated extensive BAPs and improved antioxidant activity, including lower IC₅₀ values of DPPH and ABTS radical-scavenging activities and higher FRAP. Simulated GI digestion can modify hempseed protein hydrolysates by further releasing LMW peptides, including hydrophobic sequences, which may contribute to antioxidant activity, anti-inflammatory potential, and absorption-related behavior across the intestinal barrier (11, 41). Conversely, bioactivity can decrease when active sequences are further cleaved, as reported for ACE-inhibitory chickpea and soy peptides and for tomato and orange seed protein hydrolysates, where several peptides showed reduced or lost activity after simulated GI digestion (27). Therefore, post-digestion activity depends on both peptide release and resistance to excessive cleavage (10, 16, 81). For hempseed-derived BAPs, changes in peptide composition, solubility, and resistance to extensive hydrolysis influence post-digestion bioactivity, bioaccessibility, epithelial transport, bioavailability (16, 48, 81).

Given the challenges associated with *in vivo* digestion studies, including sampling difficulty, high costs, and biological variability, standardized IVD models are widely used to reproduce gastric and intestinal digestion under controlled conditions (24, 48, 81). Biphasic and triphasic digestion models commonly use gastric digestion with pepsin at ~pH 3, followed by intestinal digestion with pancreatic and brush-border enzymes at ~pH 7 (48, 81). Static models, including the standardized INFOGEST protocol, mimic oral, gastric, and intestinal phases by controlling digestive enzymes, pH, bile salts, electrolyte composition, and digestion time (24, 81). The simulated IVD model allow controlled assessment of how digestive conditions affect protein breakdown, peptide release, further hydrolysis, and peptide resistance against digestive enzymes under physiologically relevant conditions (24, 48, 81).

Despite their standardization and reproducibility, static digestion models have notable limitations: they do not replicate physical digestive processes such as shearing, mixing, gradual hydration, peristaltic motion, or the continuous change in pH and enzyme concentration over time, instead treating each digestive phase as a fixed, uniform condition (97). Nevertheless, static protocols such as INFOGEST remain the current de facto standard for community-wide digestion screening, owing to their reproducibility, low cost, and ease of standardization across laboratories. Dynamic *in vitro* digestion systems, including the TNO gastrointestinal model (TIM-1), the Dynamic Gastric Model (DGM), the Human Gastric Simulator, and the Dynamic *In Vitro* Human Stomach (DIVHS), address these limitations by incorporating mechanical forces, peristalsis, and continuously evolving digestive conditions that are more closely approximate *in vivo* digestion (98). Direct comparisons between static and dynamic models have demonstrated meaningful physiological differences: dynamic digestion of cereal grains produced smaller particle fragments, a substantially larger chyme-enzyme contact area (451.2 vs. 160.4 cm^2^), and higher average intragastric pressure (25.0 vs. 7.2 kPa) than static digestion of the same matrix, with these differences translating into distinct downstream transcriptional responses in intestinal Caco-2 cells (99). Although dynamic digestion models have not yet been applied to hempseed-derived peptides specifically, these findings suggest that static digestion protocols may underestimate the mechanical breakdown, and consequently the peptide release and bioaccessibility, that would occur under more physiologically representative dynamic conditions, representing an important direction for future hempseed BAP research.

#### 2.4.1 Hempseed peptide-gut microbiota bidirectional interactions

Beyond small intestinal digestion, undigested hempseed protein fragments and resistant peptides that reach the colon may interact bidirectionally with the gut microbiota. Fermentation of hemp bran, a hempseed processing byproduct, by lactic acid bacteria has been shown to substantially increase short-chain fatty acid (SCFA) production, including a marked increase in butyric acid (76). SCFAs such as butyrate are established regulators of intestinal immune homeostasis, suppressing LPS-induced NF-κB activation in colonic epithelial cells via histone deacetylase inhibition and GPR109A signaling, linking colonic microbial fermentation to the same inflammatory pathway discussed in Section 2.2 (100). Consistent with this, fermented hemp seed bran has been shown to modulate gut microbiota composition and alleviate systemic autoimmune inflammation in a collagen-induced arthritis mouse model indicating that hemp-derived substrates can influence inflammatory outcomes via microbiota-mediated mechanisms rather than direct peptide bioactivity alone. However, evidence specifically demonstrating microbiome-mediated transformation of hempseed-derived peptides into new bioactive metabolites, as distinct from fiber/matrix fermentation, has not yet been reported, representing a clear gap for future hempseed peptide-microbiome research.

### 2.5 Bioaccessibility and bioavailability

After IVD, the resulting digested fraction can be evaluated for bioaccessibility, epithelial transport, bioavailability, and retained bioactivity (16, 27, 36). Bioaccessibility refers to the fraction released from the food matrix during digestion and made available for absorption in the small intestine, whereas bioavailability refers to the fraction absorbed and available at the site of action, either locally in intestinal tissues or systemically (16, 27, 46). Peptides can cross the intestinal brush-border membrane into the bloodstream via one or more of four established routes: carrier-mediated transport through peptide transporter 1 (PepT1), which favors small, hydrophobic, neutrally-charged peptides; paracellular diffusion through tight junctions, which favors hydrophilic peptides; transcytosis; and passive transcellular diffusion (101). For hempseed-derived peptides specifically, the ACE-inhibitory peptide WYT has been shown to cross Caco-2 cell monolayers via the PepT1 route in an energy-dependent manner, rather than via paracellular or transcytotic pathways (102). Not all bioactive effects require systemic absorption, as non-absorbed compounds may act locally in the GIT, for example by modulating microbiota, binding bile acids, or influencing intestinal inflammation (18). Digested fractions can be evaluated using complementary methods such as dialysis, membrane diffusion, or intestinal epithelial transport systems (16, 26, 27); among these, differentiated Caco-2 monolayers are widely used to evaluate movement across the intestinal barrier and permeability of food-derived peptides and BACs (16, 26, 27). These cells develop enterocyte-like features, including tight junctions and polarized apical and basolateral compartments, allowing estimation of apical-to-basolateral transport (27). In peptide studies, the basolateral fraction collected after Caco-2 transport can be tested for retained antioxidant or anti-inflammatory activity, indicating whether transported fractions remain biologically active rather than simply crossing the monolayer (16, 27). Nevertheless, Caco-2 cells do not fully reproduce mucus secretion, immune-cell interactions, microbiota metabolism, or the complete intestinal barrier. Co-culture systems, such as Caco-2/HT29-MTX or Caco-2/immune-cell models, may therefore provide more physiologically relevant information on epithelial and immune responses (26, 27).

Additional complementary models can provide further information on intestinal permeability and bioavailability (26). The parallel artificial membrane permeability assay estimates passive diffusion across artificial lipid membranes but cannot account for active transport, peptide degradation, paracellular transport, or cellular metabolism (24, 26, 27). *Ex vivo* intestinal models, including intestinal sacs and Ussing chamber systems, use isolated intestinal tissue to evaluate mucosal permeability, epithelial integrity, and compound movement across native intestinal barriers; however, they are technically demanding and have limited viability time (26, 27). *In vivo* studies and human intervention trials provide stronger physiological evidence but are more expensive, time-consuming, and less suitable for early screening (24, 27).

Retained bioactivity after digestion or epithelial transport is commonly evaluated using chemical and cell-based assays (11, 29). Antioxidant activity can be assessed using various *in vitro* assays such as DPPH (radical scavenging activity), TEAC (reducing power), FRAP (electron-transfer capacity), ORAC, and metal-chelating assays. Since chemical assays do not fully represent cellular metabolism, cell-based oxidative stress models are useful for determining whether digested or transported fractions reduce intracellular ROS or protect cells from oxidative injury (11). Anti-inflammatory activity is frequently evaluated using LPS-stimulated RAW 264.7 macrophages or human monocyte/macrophage models by measuring nitric oxide production, inducible nitric oxide synthase activity, and pro-inflammatory cytokines, including IL-6, TNF-α, and IL-1β (16, 29).

Collectively, the evidence reviewed above indicates that hempseed-derived bioactivity is not merely a laboratory artifact of undigested protein but is retained, and in some cases enhanced, following simulated digestion and epithelial transport. Bollati et al. (11) confirmed retained antioxidant activity for hempseed peptides H2 and H3 following Caco-2 transepithelial transport, while Zhang et al. (41) reported improved antioxidant activity following simulated GI digestion of hempseed protein. Similarly, the immunomodulatory hempseed oligopeptides characterized by Millan-Linares et al. (92) were identified specifically from Caco-2-transported fractions, with anti-inflammatory activity and TLR4/MD2 docking interactions subsequently confirmed in human monocytes, directly linking bioavailability to the inflammatory mechanism detailed in Section 2.2. The hypocholesterolemic peptide H3 characterized by Li et al. (96) is itself a transepithelial-transported peptide, indicating that its HMG-CoAR/SREBP-2/LDLR-mediated mechanism operates on a bioavailable, post-transport peptide form rather than an undigested precursor. The antihypertensive activity of hempseed peptides similarly extends beyond *in vitro* characterization: while Samaei et al. (82) identified ACE-inhibitory peptides directly from hemp bran hydrolysate, Samsamikor et al. (95) demonstrated that hemp seed protein and its hydrolysate lowered blood pressure in a human clinical trial, providing the strongest available evidence that hempseed BAP bioactivity is retained through full physiological digestion and absorption, not merely predicted from *in vitro* assays. Together, these findings support a consistent structure–function-bioavailability relationship across antioxidant, anti-inflammatory, antihypertensive, and hypocholesterolemic bioactivity classes, satisfying the nutraceutical translation objective of this review by demonstrating that mechanistically characterized hempseed BAPs remain active in physiologically relevant, post-digestion forms, with the Samsamikor human trial data representing the clearest translational confirmation to date.

#### 2.5.1 Encapsulation and delivery technologies

Encapsulation technologies, including nanoparticle, microparticle, and hydrogel-based delivery systems, have been developed to protect bioactive peptides against gastrointestinal degradation and improve their functional stability for nutraceutical applications (103). For hempseed-derived peptides specifically, Lammi et al. (104) demonstrated that native hempseed protein hydrolysates lost dipeptidyl peptidase-IV (DPP-IV) inhibitory activity when incubated in human serum, reflecting their vulnerability to proteolytic degradation. When the same hydrolysates were encapsulated within a self-assembling RADA16 peptide-based hydrogel, DPP-IV inhibitory activity was enhanced 2.5-fold and 2.0-fold in Caco-2 cells relative to the free hydrolysates, and the encapsulated hydrogels retained substantial inhibitory activity (36–37%) in human serum where the native hydrolysates showed none (104). The hydrogel additionally enabled slow, sustained peptide release over several hours and supported co-delivery with a pharmaceutical DPP-IV inhibitor, illustrating a potential strategy for combined nutraceutical-pharmaceutical formulations. Although this proof-of-concept study demonstrated encapsulation feasibility specifically for DPP-IV inhibitory (anti-diabetic) activity, direct published encapsulation data evaluating hempseed peptide antioxidant or anti-inflammatory endpoints are still absent from the literature. Nevertheless, the study provides direct evidence that hempseed-derived peptides can be structurally protected from gastrointestinal and serum proteolysis using food-grade, biocompatible delivery systems, supporting the broader industrial feasibility of hempseed BAP-based nutraceutical development discussed in the following section, though this feasibility remains to be directly demonstrated for the antioxidant, anti-inflammatory, antihypertensive, and hypocholesterolemic bioactivities emphasized in Sections 2.2 and 2.3.

### 2.6 Current market status, technical challenges, and future perspectives

The global market for alternative and plant-based proteins, including hempseed-derived ingredients, has grown substantially, with the broader alternative protein market projected to exceed USD 190 billion by 2028 at a compound annual growth rate of 18.5% between 2021 and 2027 (105). In the United States, the FDA completed evaluation of three Generally Recognized as Safe (GRAS) notices for hemp seed-derived ingredients in December 2018; hulled hemp seed, hemp seed protein powder, and hemp seed oil, raising no objections to their use in human food (106). However, these GRAS determinations cover whole hemp seed and protein powder; they do not explicitly extend to enzymatically-derived peptide fractions or hydrolysates, representing an unresolved regulatory gap directly relevant to the commercialization of the hempseed BAPs discussed above. In the European Union, hemp seed, hemp flour, and hempseed oil are explicitly exempted from Novel Food status under Regulation (EU) 2015/2283 owing to their established history of consumption; by contrast, extracts containing cannabinoids require full Novel Food safety dossier authorization (107). Whether concentrated peptide fractions or hydrolysates derived from hempseed protein would fall under this exemption or instead trigger novel-process provisions of the regulation remains an open regulatory question that has not been directly addressed in the literature. Across both jurisdictions, therefore, existing GRAS status and Novel Food exemptions apply specifically to whole hempseed material rather than to purified, enzymatically-derived peptide hydrolysate fractions, leaving the regulatory status of hempseed BAPs themselves unresolved. Internationally, inconsistent THC threshold regulations and import requirements across jurisdictions have caused border delays, import restrictions, and unpredictable market access for hemp-derived food products more broadly (108).

Beyond regulatory considerations, technical challenges limit industrial translation of hempseed BAPs. Hempseed protein and peptide yields vary considerably by cultivar, growing conditions, and extraction method, complicating standardization of commercial ingredients (19, 21–23, 35). As discussed throughout the preceding sections, enzymatic hydrolysis and fractionation processes have been characterized primarily at laboratory scale; hempseed-specific data on reproducibility, cost, or yield consistency at industrial production scale have not been reported in the literature and represent a clear knowledge gap. Similarly, encapsulation and delivery technologies to protect hempseed BAPs during gastrointestinal transit, an established strategy in other bioactive peptide systems, have not yet been investigated specifically for hempseed-derived peptides.

Future research should prioritize: (1) hempseed-specific dose–response characterization to establish safe and effective concentration ranges for nutraceutical use; (2) direct structural evidence of hempseed peptide interactions with intracellular targets such as NF-κB, complementing existing receptor-level (TLR4/MD2) docking data; (3) investigation of microbiome-mediated transformation of hempseed peptides in the colon, an area with no direct hempseed-specific evidence to date; (4) dynamic, physiologically representative GI digestion models to complement current static protocols; and (5) regulatory clarification of whether hempseed-derived peptide fractions and hydrolysates fall within existing GRAS and Novel Food frameworks established for whole hemp seed ingredients. Addressing these scientific and regulatory priorities will be necessary to translate hempseed-derived BAPs from laboratory findings into viable commercial nutraceutical and functional food ingredients.

Human evidence for hempseed BAP bioactivity remains critically limited. To date, the only human intervention study is the double-blind, randomized crossover trial by Samsamikor et al. (95), which evaluated hemp seed protein and its hydrolysate against blood pressure endpoints in adults with mild hypertension. Beyond this single trial, no published human intervention studies have examined antioxidant or anti-inflammatory endpoints for hempseed-derived bioactive peptides; the antioxidant, anti-inflammatory, and hypocholesterolemic mechanisms detailed throughout this review are supported exclusively by *in vitro* (cell-based) and simulated digestion/transport evidence. This absence of human antioxidant/anti-inflammatory intervention data represents the most critical translational bottleneck for advancing hempseed-derived BAPs toward evidence-based nutraceutical or functional food applications, and should be prioritized in future clinical research.

## 3 Conclusion

Hempseeds are a promising plant-based source of nutrients and bioactive compounds, including bioactive peptides with antioxidant, anti-inflammatory, antihypertensive, and hypocholesterolemic properties mediated through defined molecular mechanisms rather than general peptide bulk properties. Bioactivity depends on processing-induced changes in amino acid profile, peptide release, structure, and gastrointestinal stability, as well as concentration-dependent effects relevant to nutraceutical dosing safety. This review integrated structure–function relationships across bioactivity classes and identified key gaps including the absence of hempseed-specific dose–response data, direct structural evidence for peptide-NF-κB interactions, microbiome-mediated peptide metabolism, and industrial-scale processing and regulatory clarity that must be addressed to translate hempseed-derived peptides into viable functional food and nutraceutical applications.
